# Supplementary material for: Reconciling Mining with the Conservation of Cave Biodiversity: A Quantitative Baseline to Help Establish Conservation Priorities
Source: PLoS One. 2016 Dec 20;11(12):e0168348. doi: 10.1371/journal.pone.0168348 (PMC5173368; doi:10.1371/journal.pone.0168348)
Supplement: S1 Dataset — (ZIP) [file pone.0168348.s002.zip › Taxa/Serra Norte/SN_2013/N4WS/Lista N4WS-12.pdf]

CAVIDADE N4WS-0012

Classe	Ordem	Fam/Outros	Gên/Outros	Espécie	S-U	U-S	Zon.
Arachnida	Amblypygi	Charinidae	*Charinus*	sp.1		7	E/P
Arachnida	Amblypygi	Phrynidae	*Heterophrynus*	*longicornis*	X	2	E/P
Arachnida	Araneae	Araneidae		jovem	X	X	P
Arachnida	Araneae	Ctenidae		jovem		1	E
Arachnida	Araneae	Ochyroceratidae	*Speocera*	sp.1	X	X	P
Arachnida	Araneae	Pholcidae	Mesabolivar	*Aurantiacus*		X	
Arachnida	Araneae	Salticidae		sp.	X		
Arachnida	Araneae	Scytodidae	*Scytodes*	*eleonorae*	1		
Arachnida	Araneae	Scytodidae	*Scytodes*	*globula*		1	E
Arachnida	Araneae	Theridiidae	*Theridion*	sp.	X		
Arachnida	Araneae	Theridiosomatidae	*Plato*	sp.	X	X	
Arachnida	Opiliones	Escadabiidae		sp.2		X	E
Arachnida	Pseudoscorpiones	Chernetidae	*Spelaeochernes*	sp.1	X	X	P
Arachnida	Pseudoscorpiones	Chthoniidae	*Pseudochthonius*	sp.1		X	P
Arachnida	Pseudoscorpiones	Chthoniidae		sp.	X		
Arachnida	Schizomida	Hubbardiidae	aff. *Rowlandius*	sp.1	X		
Chilopoda	Scutigeromorpha			jovem	X		
Diplopoda	Polydesmida	Pyrgodesmidae		sp.1	X	X	
Diplopoda	Spirostreptida	Pseudonannolenidae	*Pseudonannolene*	jovem		2	P
Entognatha	Collembola	Isotomidae		sp.1		X	E
Entognatha	Collembola			sp.	X		
Entognatha	Diplura	Projapygidae		sp.1		X	E
Insecta	Blattodea			jovem	X		
Insecta	Coleoptera	Carabidae		sp.	X		
Insecta	Coleoptera	Staphylinidae		jovem	X		
Insecta	Diptera	Culicidae		sp.	X		
Insecta	Diptera	Phoridae		sp.	X		
Insecta	Diptera	Psychodidae	Phlebotominae	sp.		X	P
Insecta	Diptera	Psychodidae	*Pintomyia*	*gruta*	X		
Insecta	Diptera	Psychodidae	*Sciopemyia*	*sordellii*	X		
Insecta	Diptera	Tipulidae		sp.	X		
Insecta	Heteroptera	Cydnidae		sp.		X	P
Insecta	Heteroptera	Dipsocoroidea		sp.		1	E
Insecta	Heteroptera	Reduviidae	*Zelurus*	sp.	X		
Insecta	Homoptera	Cixiidae		jovem	X		
Insecta	Hymenoptera	Braconidae		sp.1		X	
Insecta	Hymenoptera	Formicidae	*Camponotus*	sp.1		X	E
Insecta	Hymenoptera	Formicidae	*Pheidole*	sp.2		X	P
Insecta	Hymenoptera	Formicidae	*Solenopsis*	sp.1		X	P
Insecta	Isoptera	Termitidae		sp.	X		
Insecta	Lepidoptera	Noctuoidea		jovem	X		
Insecta	Lepidoptera	Tineoidea		jovem	X		
Insecta	Orthoptera	Phalangopsidae	*Paraclodes*	sp.	X		
Insecta	Orthoptera	Phalangopsidae	*Phalangopsis*	sp.	X	62	E/P
Insecta	Psocoptera	Epipsocidae	*Mesepipsocus*	sp.1	X	X	P
Gastropoda	Pulmonata	Subulinidae		sp.2		X	E
Malacostraca	Isopoda	Armadillidae		sp.1	X	X	P
Malacostraca	Isopoda	Philosciidae		sp.1		X	E
Malacostraca	Isopoda	Scleropactidae		sp.1		X	P

Amphibia	Anura	Ranidae		sp.		1	P
Amphibia	Anura			sp.		2	E
Mammalia	Chiroptera	Phyllostomidae		sp.	10		E/P
